# Approximation of Densities on Riemannian Manifolds

**DOI:** 10.3390/e21010043

**Published:** 2019-01-09

**Authors:** Alice le Brigant, Stéphane Puechmorel

**Affiliations:** Ecole Nationale de l’Aviation Civile, Université de Toulouse, 31055 Toulouse, France

**Keywords:** quantization, directional densities, exponential family, group invariance, Riemannian manifold

## Abstract

Finding an approximate probability distribution best representing a sample on a measure space is one of the most basic operations in statistics. Many procedures were designed for that purpose when the underlying space is a finite dimensional Euclidean space. In applications, however, such a simple setting may not be adapted and one has to consider data living on a Riemannian manifold. The lack of unique generalizations of the classical distributions, along with theoretical and numerical obstructions require several options to be considered. The present work surveys some possible extensions of well known families of densities to the Riemannian setting, both for parametric and non-parametric estimation.

## 1. Introduction

In probability and statistics, random variables whose law admits a probability density (with respect to e.g., the Lebesgue measure) are more tractable than general ones, both from the theoretical and the algorithmic point of view. When dealing with experimental data, the density is generally unknown and must be estimated.

In many cases, it is a function belonging to a given family, defined on the image of the random variable. When the family depends on a finite number of parameters, the estimation problem boils down to finding a point in the parameter space minimizing a goodness-of-fit criterion. Methods pertaining to this class are referred to as parametric. On the other hand, when prior information on the true density is lacking or when the parametric approach is too complicated or expensive from a computational point of view, it may be more pertinent to use another class of methods, the *non-parametric* ones, that does not rely on fitting parameters. In this case, the true density is computed from the samples themselves, often by summing copies of a model density known as a kernel function.

On the other hand, some applications require an approximation rather than a proper estimation of the density of a given dataset. When dealing with large datasets, it can be interesting to search for a summary of the empirical distribution in the form of a discrete probability measure with a given number of supporting points. This is known as the quantization problem and has received much attention from the signal processing community. It is worth noticing that finding an optimal quantization is closely related to clustering: each supporting point in the discrete distribution can be thought as a cluster center, with a membership function that associates with a sample the closest supporting point.

In all the above cases, density approximation is central. When the considered random variables are defined on a finite dimensional real vector space, the problem has been extensively studied [1,2]. However, in many applications, the data are best modeled as elements of a Riemannian manifold, and approximation procedures have to be adapted. Classical examples include the sphere $S^{d}$, the *d*-dimensional torus and some matrix spaces. In medical imaging, for example, diffusion tensor images have pixels taking their values in the cone of symmetric positive definite matrices. The same type of data arises when assessing a complexity level to a traffic situation in the air transportation system context. The geometry of correlation matrices is related to the hyperbolic space and is very informative in signal processing applications. An important issue is the lack of a unique extensions of commonly used distributions, like the Gaussian one. The problem is even more acute if one wants to use these model distributions as elementary bricks for approximating more complex ones. This paper will survey the different options offered to estimate and approximate probability densities on Riemannian manifolds. After a brief summary of the main notions of Riemannian geometry that will be needed in the sequel, we present parametric estimation in Section 3. Section 4 and Section 5 are devoted to non-parametric estimation, and Section 6 to optimal quantization.

## 2. Some Notions from Riemannian Geometry

### 2.1. Differentiable Manifolds

A *topological manifold* of dimension *d* is a topological space *M* that can be locally approximated at any point p∈M by a subset of $R^{d}$ through a so-called *local chart*, that is, a homeomorphism $\varphi :U\to R^{d}$ (a continuous, bijective map with continuous inverse) defined on a neighborhood *U* of *p*. A collection of local charts ${\left(U_{i},\varphi_{i}\right)}_{i\in I}$ such that the union of the $U_{i}$’s covers all of *M* is called an *atlas*, and *M* is said to be *differentiable* if one can go from one local chart to another in a differentiable way. More precisely, *M* is of class $C^{k}$ if the *transition maps* $\varphi_{i}\circ \varphi_{j}^{-1}$ defined by composing chart maps with intersecting domains $U_{i}\cap U_{j}\neq \emptyset$ are $C^{k}$ maps from $\varphi_{j}\left(U_{i}\cap U_{j}\right)\subset R^{d}$ to $\varphi_{i}\left(U_{i}\cap U_{j}\right)\subset R^{d}$. Moreover, if the Jacobian determinants of the transition maps are positive, the manifold is said to be *orientable*. This property is mandatory to define a volume form, an object that is used repeatedly in the sequel to define densities. However, in case of non-orientable manifolds, it is still possible to use the weaker notion of Riemannian density. It is an odd-type differential form in the sense of [3]. In the rest of the paper, for the sake of simplicity, we will always consider smooth ($C^{\infty }$), orientable manifolds.

The local charts allow us to transpose (local) computations to the familiar Euclidean framework and to export definitions from that setting. Given another differentiable manifold *N* of dimension *n*, we can naturally define a mapping F:M→N to be differentiable at a point p∈M if a given (or equivalently, any) representation $\psi \circ F\circ \varphi^{-1}$ of *F* using local charts $\varphi :U\to R^{d}$ of *M* and $\psi :V\to R^{n}$ of *N* is differentiable at ϕ(p) as a map from $\varphi^{-1}\left(U\right)\subset R^{d}$ to $R^{n}$. In what follows, we may place ourselves in a local chart (U,φ) and use the corresponding local coordinates $(x_{1}\left(p\right),\ldots ,x_{d}\left(p\right)):=\varphi \left(p\right)$.

### 2.2. Tangent and Cotangent Vectors

A *tangent vector* at a point *p* can be seen as the intrinsic (i.e., compatible with chart transition functions) velocity of a curve in *M* passing through *p*, as well as a derivation acting on the algebra $F_{p}$ of germs at *p* of smooth real-valued functions f:M→R [4] (Chapter 1.7).

More precisely, let α:(−ϵ,ϵ)→M be a smooth curve on *M*, and let p=α(0). For any smooth function f:M→R with germ $\left[f\right]\in F_{p}$, the derivative

$${\left.\frac{d}{dt}\right|}_{t=0}f\circ \alpha \left(t\right)$$

depends only on [f] and will be denoted as X([f]). The operator $X:F_{p}\to R$ is obviously linear and satisfies the Liebniz rule:

$$X\left([fg]\right)=X\left(\left[f\right]\right)\left[g\right]\left(p\right)+\left[f\right]\left(p\right)X\left(\left[g\right]\right).$$

*X* is called the tangent vector to α at *p*. Using Hadamard’s lemma in a chart, one can show that *X* can be in fact represented by a vector in $R^{d}$, hence the name. Please note that *X* as a derivation is coordinate-free, while it depends on the local coordinates when represented in $R^{d}$.

A tangent vector at *p* is a tangent vector to a certain curve passing through *p* at time zero. The set of all tangent vectors at *p* defines the *tangent space* $T_{p}M$, a vector space of same dimension as *M*, and the collection of all tangent spaces defines the *tangent bundle* $TM=\cup_{p\in M}T_{p}M$. Given a coordinate chart $\varphi =(x_{1},\ldots ,x_{d})$, the tangent vectors defining partial derivation according to coordinates $x_{i}$ are denoted by $\frac{\partial }{\partial x_{1}}\left(p\right),\ldots ,\frac{\partial }{\partial x_{d}}\left(p\right)$ and define a basis of $T_{p}M$. As any vector space, the tangent space at *p* admits a dual space $T_{p}^{*}M$ called the *cotangent space*, and composed of linear forms $z_{p}:T_{p}M\to R$, also called *cotangent vectors*. The basis of $T_{p}M^{*}$ in local coordinates is denoted by $dx_{1}\left(p\right),\ldots ,dx_{d}\left(p\right)$.

### 2.3. Pullback and Pushforward

Associated with the dual notions of tangent and cotangent vectors are the dual notions of *pushforward* and *pullback*. Given a smooth map F:M→N between two smooth manifolds, the pushforward of *F* is a linear map $F_{*}:TM\to TN$ that maps tangent vectors $X_{p}$ at point p∈M to tangent vectors $F_{*}X_{p}$ at the image point F(p)∈N. The vector $F_{*}X_{p}$ can be defined as acting on real-valued functions f:N→R or equivalently, as the velocity vector of a curve α:(−ϵ,ϵ)→M passing through *p* at time zero with speed $\dot{\alpha }\left(0\right)=X_{p}$,

$$\left(F_{*}X_{p}\right)\left(f\right)=X_{p}\left(f\circ F\right),\;F_{*}X_{p}={\left.\frac{d}{dt}\right|}_{t=0}F\circ \alpha \left(t\right).$$

The pushforward is also called the *differential* of *F* and can also be denoted $d_{p}F:={\left(F_{*}\right)}_{p}$. Symmetrically, the pullback maps cotangent vectors $z_{F(p)}$ at F(p)∈N to cotangent vectors at p∈M, acting on tangent vectors $X_{p}\in T_{p}M$ as

$$\left(F^{*}z_{F(p)}\right)\left(X_{p}\right)=z_{F(p)}\left(F_{*}X_{p}\right).$$

### 2.4. Vector Fields and Covariant Derivatives

A *vector field* is a mapping X:M→TM that associates with each point *p* a tangent vector $X_{p}\in T_{p}M$. Just as tangent vectors, it acts on differentiable functions f:M→R in a way that can be written, in local coordinates, as

$$X_{p}\left(f\right)=\sum_{i=1}^{d}a_{i}\left(p\right)\frac{\partial f}{\partial x_{i}}\left(p\right).$$

It is possible to take the derivative of a vector field with respect to another using an *affine connection*, that is, a functional ∇ that acts on pairs of vector fields $\left(X,Y\right)\mapsto \nabla_{X}Y$ according to the following rules

$$\begin{array}{c} \nabla_{fX+Y}Z=f\nabla_{X}Z+\nabla_{Y}Z,\\ \nabla_{X}\left(fY\right)=X\left(f\right)Y+f\nabla_{X}Y,\;\nabla_{X}\left(Y+Z\right)=\nabla_{X}Y+\nabla_{X}Z. \end{array}$$

This action is referred to as *covariant derivative*. Vector fields V:(−ϵ,ϵ)→TM can also be defined along a curve α(t), that is, V(t) is an element of $T_{\alpha (t)}M$) for all *t*. Then, covariant derivative is sometimes denoted by $\frac{DV}{dt}:=\nabla_{\dot{\alpha }\left(t\right)}V$.

### 2.5. Riemannian Metric and Geodesics

The possibility to compute angles and lengths in a differentiable manifold is given by a *Riemannian metric*, i.e., a smoothly varying inner product $g_{p}:T_{p}M\times T_{p}M\to R$ defined on each tangent space $T_{p}M$ at p∈M (recall that $T_{p}M$ is a vector space). The subscript *p* in the metric will often be omitted and the associated norm will be denoted by ∥·∥:=g(·,·). There is only one affine connection that is symmetric, meaning $XY-YX=\nabla_{X}Y-\nabla_{Y}X$, and compatible with the Riemannian metric, which is

$$\frac{d}{dt}g\left(U,V\right)=g\left(\frac{DU}{dt},V\right)+g\left(U,\frac{DV}{dt}\right),$$

for any vector fields U,V along a curve α. It is called the *Levi–Civita connection* associated with *g* and will be denoted by ∇ from now on. Just as the Euclidean distance can be measured as the length of a straight line, distances in a Riemannian manifold are computed through the length of minimizing *geodesics*. The geodesics of *M* are the curves γ satisfying the relation $\nabla_{\dot{\gamma }}\dot{\gamma }=0$, which implies that their speed has constant norm $∥\dot{\gamma }\left(t\right)∥=cst$. They are also the local minimizers of the arc length functional *l*:

$$l:\gamma \mapsto \int_{0}^{1}∥\dot{\gamma }\left(t\right)∥dt$$

if curves are assumed, without loss of generality, to be defined over the interval [0,1]. When it exists, the length of the shortest geodesic linking two points defines their geodesic distance. The *cut locus* of p∈M is the set of points where the geodesics starting at *p* stop being minimizing, and the *injectivity radius* at *p* is its distance to the cut locus. The global injectivity radius of the manifold is the infimum of the injectivity radii over all points of *M*.

### 2.6. Exponential and Logarithm Maps

From the geodesics of *M*, we can now define the *exponential map* at point *p*, a diffeomorphism (i.e., a differentiable, bijective map of differentiable inverse) denoted by $\exp_{p}$, which maps a tangent vector *v* of an open ball $B\left(0,r\right)\subset T_{p}M$ centered in 0 to the endpoint $\gamma \left(1\right)=:\exp_{p}\left(v\right)$ of the geodesic γ:[0,1]→M verifying γ(0)=p, $\dot{\gamma }\left(0\right)=v$. Intuitively, the exponential map moves the point *p* along the geodesic starting from *p* at speed *v* and stops after covering the length ∥v∥. Conversely, the inverse of the exponential map $\log_{p}\left(q\right):=\exp_{p}^{-1}\left(q\right)$ gives the vector that maps *p* to *q*. The image by the exponential map of the open ball $B\left(0,r\right)\subset T_{p}M$, with *r* less than the injectivity radius at *p*, is called the *geodesic ball* of radius r>0 centered in *p*.

### 2.7. Curvature and Jacobi Fields

The *curvature tensor* of *M* associates with any pair of vector fields (X,Y) on *M* a linear mapping R(X,Y) on the space of vector fields, defined for all vector field *Z* by

$$R\left(X,Y\right)Z:=\nabla_{X}\nabla_{Y}Z-\nabla_{Y}\nabla_{X}Z-\nabla_{\left[X,Y\right]}Z,$$

where [X,Y]:=XY−YX denotes the *Lie bracket*. Another way to characterize the curvature of *M* is through the *sectional curvature*, which is defined for any two-dimensional subspace $\sigma \subset T_{p}M$ of the tangent space at *p* by

$$K\left(\sigma \right)=\frac{g_{p}\left(R\left(u,v\right)v,u\right)}{g_{p}\left(u,u\right)g_{p}\left(v,v\right)-g_{p}{\left(u,v\right)}^{2}},$$

if u,v are two linearly independent vectors that span σ. Due to the curvature of a manifold, geodesics spreading out from a given point *p* can either diverge (negative curvature) or converge (positive curvature). The way these geodesics spread out is described by the *Jacobi fields*. If $\left\{t\mapsto \exp_{p}\left(tv\left(s\right)\right),s\in \left(-\epsilon ,\epsilon \right)\right\}$ is a sheave of geodesics starting from the same point *p* at speeds $v\left(s\right)\in T_{p}M$, and $\gamma \left(t\right):=\exp_{p}\left(tv\left(0\right)\right)$ denotes the one at the center of the sheave, the vector field along γ defined for all *t* by

$$J\left(t\right)={\left.\frac{d}{ds}\right|}_{s=0}\exp_{p}\left(tv\left(s\right)\right)$$

is a Jacobi field along γ. It is the only one with initial conditions J(0)=v(0) and $\frac{DJ}{dt}\left(0\right)=\dot{v}\left(0\right)$ (where we identify the two vector spaces $T_{p}M\approx T_{v(0)}T_{p}M$) verifying the Jacobi equation $\frac{D^{2}}{dt^{2}}J+R\left(J,\dot{\gamma }\right)\dot{\gamma }=0$.

### 2.8. Measures and Integration over a Riemannian Manifold

A *differentiable k-form* ω on an orientable *d*-dimensional manifold *M* associates with all p∈M an alternating multilinear function $\omega_{p}:{\left(T_{p}M\right)}^{d}\to R$ (i.e., $\omega_{p}$ associates zero to any *d*-tuple with a repetition). If ω is a differentiable *k*-form on a manifold *N*, then any smooth map F:M→N induces by pullback a *k*-form on *M* acting on *k*-tuples of tangent vectors at p∈M as

$${\left(F^{*}\omega \right)}_{p}\left(u_{1},\ldots ,u_{k}\right)=\omega_{F(p)}\left(F_{*}u_{1},\ldots ,F_{*}u_{k}\right).$$

The *volume forms* of *M* are the differential forms of maximal degree *d* (the dimension of the manifold), and are the only ones that can be integrated over *M*. If (U,φ) is a local chart such that suppω⊂U, then ${\left(\varphi^{-1}\right)}^{*}\omega$ is a *d*-form on $R^{d}$, and so it admits a density $f:\varphi^{-1}\left(U\right)\mapsto R$ with respect to the volume element defined in local coordinates by the exterior product $dx_{1}\wedge \ldots \wedge dx_{d}$. The integral of the volume form ω is then defined by

$$\int_{U}\omega :=\int_{\varphi^{-1}\left(U\right)}{\left(\varphi^{-1}\right)}^{*}\omega =\int_{\varphi^{-1}\left(U\right)}f\left(x\right)dx.$$

Every volume form defines a measure on *M*, which is written by extension in local coordinates $d\mu =fdx_{1}\wedge \ldots \wedge dx_{d}$ or dμ=fdx for short. The *Riemannian measure* is the volume form which density is given by the square root of the determinant of the Riemannian metric, i.e.,

$$d\mathrm{vol}\left(x\right)=\sqrt{\det G(x)}dx,$$

where G(x) is the d×d matrix with entries

$$G_{ij}\left(x\right)=g_{x}\left(\frac{\partial }{\partial x_{i}}\left(x\right),\frac{\partial }{\partial x_{j}}\left(x\right)\right).$$

The Riemannian measure will play the role of the Lebesgue measure for integrals defined on *M*.

### 2.9. The Laplace–Beltrami Operator

Finally, in order to make this work self-contained, we introduce the generalization of the Laplacian to manifolds, namely, the *Laplace–Beltrami operator*. Let *X* be a vector field on *M* and $\phi_{X}\left(t,x\right)$, (t,x)∈(−ϵ,ϵ)×U its *local flow* in a neighborhood *U* of p∈M, i.e., such that for all *x*, $t\mapsto \phi_{X}\left(t,x\right)$ is the unique curve verifying $\partial_{t}\phi_{X}\left(t,x\right)=X_{\phi_{X}\left(t,x\right)}$ and $\phi_{X}\left(0,x\right)=x$. Then, the *Lie derivative* of the volume form along the vector field *X* is given by the derivative of its pullback by the flow of *X*

$$L_{X}\mathrm{vol}={\left.\frac{d}{dt}\right|}_{t=0}\phi_{X}{\left(t,\cdot \right)}^{*}\mathrm{vol}.$$

Intuitively, it measures the way infinitesimal volume is transported by *X*. Since $L_{X}\mathrm{vol}$ is a *d*-form, it admits a density with respect to the Riemannian volume form, which is defined to be the *divergence* of *X*, i.e., $L_{X}\mathrm{vol}=\left(\mathrm{div}X\right)\mathrm{vol}$. Then, the Laplace–Beltrami operator of a function f:M→R is, just as the Euclidean case, defined as the divergence of its gradient

Δf=div(gradf),

where the gradient is linked to the differential (or pushforward) as $g_{p}\left({\mathrm{grad}}_{p}f,X_{p}\right)=d_{p}f\left(X_{p}\right)$ for any tangent vector $X_{p}\in T_{p}M$.

The Laplace–Beltrami operator can defined alternatively using the Levi–Civita connection. Let X,Y be vector fields and f:M→R and f:M→R as above. The Hessian of *f* is the symmetric 2-tensor:

$$H\left(f;X,Y\right)=\nabla_{Y}\nabla_{X}f-\nabla_{\nabla_{Y}X}f.$$

The Laplacian of *f* is then defined as the trace of the Hessian with respect to the metric:

$$\Delta f=g^{ij}H\left(f;\partial_{i},\partial_{j}\right),$$

where $g^{ij}$ stands for the i,j element of the inverse metric matrix and $\partial_{i}$ is the *i*-th coordinate vector field.

## 3. Parametric Estimation

Let (E,F,μ) be a measure space. In the sequel, all distributions are assumed to be absolutely continuous with respect to μ and all densities will thus implicitly refer to it. In parametric density estimation, one wants to approximate an unknown distribution with density *f* by a member of a given parameterized family $\left\{f_{\theta }:E\to R^{+},\theta \in \Theta \right\}$ of densities. Most of the time, the optimal $\theta^{*}$ is found using a maximum likelihood procedure: if ${\left(X_{i}\right)}_{i=1\ldots N}$ is an iid sample with common distribution *f*, then

$$\theta^{*}={\mathrm{argmax}}_{\theta \in \Theta }\prod_{i=1}^{N}f\left(X_{i}|\theta \right).$$

The only requirement on the domain *E* of the family $\left\{f_{\theta },\theta \in \Theta \right\}$ is to be a measure space, which of course encompasses the Riemannian manifold case, with μ=vol, the Riemannian volume.

Being able to obtain a meaningful parameterized distribution family on a general manifold is not an easy task in general. Some clues will be given at the end of this section. In special cases, some well known distributions were introduced. Some of them will be presented now.

### 3.1. Directional Statistics

Directional statistics [5] deals with inference on unit vectors samples and introduces ad hoc distributions. Since unit vectors can be seen as points on the unit sphere of the underlying vector space, it yields a basic, yet extremely useful example of parameterized families of distributions on a Riemannian manifold.

Since the unit sphere $S^{d-1}\subset R^{d}$ has rotational invariance, it is expected that the parameterized families ${\left(f_{\theta }\right)}_{\theta \in \Theta }$ exhibit the same behavior, i.e.,

(1) $$\forall A\in \mathrm{SO}\left(d\right),\forall \theta \in \Theta ,\forall x\in S^{n},f_{A\theta }\left(Ax\right)=f_{\theta }\left(x\right).$$

Please note that, to be able to write such a covariance property, it is required to have an action of the group SO(d) on Θ. The case $\Theta =S^{d-1}$ is, up to our knowledge, the only one that has been considered by the directional statistics community.

A common choice is the von Mises–Fisher (vMF) distribution on $S^{d-1}$, denoted M(μ,κ), given by the density [6]

(2) $$f_{\lambda }\left(x;m\right)=c_{d}\left(\kappa \right)\;e^{\kappa \langle \mu ,x\rangle },\;\kappa >0,\;x\in S^{d-1},$$

where

(3) $$c_{d}\left(\lambda \right)=\frac{\lambda^{d/2-1}}{{\left(2\pi \right)}^{d/2}I_{d/2-1}\left(\lambda \right)}$$

is a normalization constant with $I_{r}\left(\lambda \right)$ denoting the modified Bessel function of the first kind at order *r*. The vMF density is unimodal, parameterized by the mean μ and the concentration parameter κ>0 that controls the dispersion of the distribution around the mean. The limiting, degenerate case κ=0 yields the uniform distribution on the sphere.

When the expectations of the projections on a fixed orthonormal basis $\left(e_{1},\ldots ,e_{d}\right)$ are given, it is a maximum entropy distribution. This fact can be easily seen by writing the associated variational problem with linear constraints:

(4) $$\left\{\begin{array}{c} {\mathrm{argmax}}_{f}\int_{S^{d-1}}f\left(x\right)\log f\left(x\right)d\sigma \left(x\right),\\ \int_{S^{d-1}}f\left(x\right)\langle e_{i},x\rangle d\sigma \left(x\right)=a_{i},\;i=1\ldots d,\\ \int_{S^{d-1}}f\left(x\right)d\sigma \left(x\right)=1, \end{array}\right.$$

with σ the solid angle measure on the sphere. Using Lagrange multipliers $\lambda_{1},\ldots \lambda_{d}$ for the first *d* constraints and *c* for the last one yields a general form for the solution:

(5) $$f\left(x;c,\lambda \right)=\exp\left(c+\langle x,\lambda \rangle \right)$$

with $\lambda =(\lambda_{1},\ldots ,\lambda_{d})$. The *c* is a normalization constant. The remaining ones can be interpreted as mean parameters by normalization, provided λ≠0:

〈x,λ〉=∥λ∥〈x,λ/∥λ∥〉.

The vMF density extends readily to the Stiefel manifold O(d,p) of *p*-dimensional orthonormal families in $R^{d}$ using the same maximum entropy approach, but using projections on the elementary matrices of dimension d×p. The general form of the distribution is then:

(6) $$f\left(X;M\right)=c\left(M\right)\exp\left(\mathrm{tr}M^{t}X\right),\;M\in M_{d,p},\;X\in O\left(d,p\right).$$

As in the spherical vMF case, *M* cannot be interpreted directly as a mean on O(d,p) and some kind of normalization is needed. In order to better understand the behavior of *M*, it is useful to use its singular values decomposition (SVD) decomposition [7]. Let $M=U\Sigma V^{t}$ with $U\in M_{d,d}$, $V\in M_{p,p}$, $\Sigma \in M_{d,p}$ and U,V orthogonal matrices. Then:

(7) $$\mathrm{tr}M^{t}X=\mathrm{tr}V\Sigma^{t}UX=\mathrm{tr}\Sigma^{t}UXV.$$

Let $C_{j}$, j=1…p, be the columns of the d×p matrix XV. It comes:

(8) $$\mathrm{tr}M^{t}X=\sum_{j=1}^{p}\sigma_{j}\langle U_{j},C_{j}\rangle ,$$

where $\sigma_{j}$ is the *j*-th diagonal element of Σ and $U_{j}$ is the *j*-th row of *U*. The net result is thus a product of vMF, after a change of basis given by the matrix *V*. A rank deficiency in the matrix *M* indicates a uniform distribution on a subspace, much like in the standard vMF case with κ=0. In the matrix case, the limiting case can occur on full subspaces.

A further extension to the Grassmann manifold can be done by quotienting out the density with respect to the O(p) group action [8].

A generalization of maximum entropy distributions with moment constraints to Riemannian manifolds *M* can be found in [9], where an analogous of the normal law is obtained. The constraints chosen are, in normal coordinates around the mean value:

(9) $$\left\{\begin{array}{c} \int f(x)d\mathrm{vol}(x)=1,\\ \int xf(x)d\mathrm{vol}(x)=0,\\ \int xx^{t}f\left(x\right)d\mathrm{vol}\left(x\right)=\Sigma , \end{array}\right.$$

where Σ is a fixed symmetric, positive definite matrix. The resulting density is parameterized by a mean μ and concentration matrix Γ and is expressed as:

(10) $$f_{\mu ,\Gamma }\left(p\right)\propto \exp\left(-\frac{\log_{\mu }{\left(p\right)}^{t}\Gamma \log_{\mu }\left(p\right)}{2}\right),\;p\in M.$$

As mentioned in [9], the distribution may not be differentiable or even continuous on the cut locus.

Finally, a different construction [10,11] yields a directional distribution on the hyperbolic *d*-dimensional space. Only the approach of [11] will be detailed here, as it introduces another way to obtain distributions on manifolds using exit points of a Brownian motion with drift. The general idea underlying this approach is to use a submanifold of a well-known model manifold on which the Brownian motion with drift can be constructed. Starting at a fixed origin, a Brownian motion path will intersect the submanifold for the first time at a point, called the exit point. The distribution of the exit points will yield a generalized directional distribution. The original motivation of this construction comes from the exit distribution of a Brownian motion with drift starting at the origin on the unit circle in $R^{2}$. The resulting density turns out to be exactly the vMF.

In the hyperbolic space, the Brownian motion is a diffusion with infinitesimal generator:

(11) $$\frac{x_{d}^{2}}{2}\left(\sum_{i=1}^{d}\frac{\partial^{2}}{\partial x_{i}^{2}}\right)-\frac{\left(d-2\right)x_{d}}{2}\frac{\partial }{\partial x_{d}},$$

where all the coordinates are given in the half-space model of $H^{d}$:

$$H^{d}=\left\{x_{1},\ldots ,x_{d}:x_{i}\in R,\;i=1,\ldots ,d-1,\;x_{d}\in R^{+}\right\}.$$

It is convenient to represent the half-space model of $H^{2}$ in C, with z=x+iy, y>0. The two-dimensional hyperboloid embedded in $R^{3}$ associated with $H^{2}$ is given by:

(12) $$\{\left(x_{1},x_{2},x_{3}\right):x_{1}^{2}+x_{2}^{2}-x_{3}^{2}=-1\}.$$

It admits hyperbolic coordinates:

(13) $$x_{1}=\sinh\left(r\right)\cos\left(\theta \right),x_{2}=\sinh\left(r\right)\sin\left(\theta \right),x_{3}=\cosh\left(r\right),$$

that transforms to the unit disk model as:

(14) $$u=\frac{\sinh(r)\cos(\theta )}{1+\cosh(r)},\;v=\frac{\sinh(r)\sin(\theta )}{1+\cosh(r)},$$

where θ and *r* are the angular and radius coordinates. Finally, using a complex representation z=u+iv and the Möbius mapping z→i(1−z)/(1+z), it comes the expression of the half-plane coordinates:

(15) $$x=\frac{\sinh(r)\sin(\theta )}{\cosh(r)+\sinh(r)\cos(\theta )},\;y=\frac{1}{\cosh(r)+\sinh(r)\cos(\theta )}.$$

The hyperbolic von Mises distribution is then defined, for a given r>0, as the density of the first exit on the circle of center *i* and radius *r* of the hyperbolic Brownian motion starting at *i*. Its expression is given in [11] (Section 2.2, Propostion 2) as:

(16) $$f_{v}\left(r,\theta \right)=\frac{1}{2\pi P_{-\nu }^{0}\left(\cosh\left(r\right)\right)}{\left(\cosh(r)+\sinh(r)\cos(\theta )\right)}^{-\nu },$$

where $P_{-\nu }^{0}$ is the Legendre function of the first kind with parameters 0,−ν, which acts as a normalizing constant to get a true probability density. The parameter ν is similar to the concentration used in the classical von Mises distribution.

### 3.2. Gaussian-Like Distributions

The maximum entropy distributions introduced above are not the only possible choice for probabilities on manifolds. Another approach may be to mimic the multivariate normal density using the geodesic distance on the manifold. For the space of symmetric positive definite matrices, one can refer to [12], and to [13] for the general case of symmetric spaces.

Let *M* be a symmetric space [14]. The Gaussian-like density on *M* with mean μ and variance $\sigma^{2}>0$ is given by:

(17) $$f_{\mu ,\sigma }\left(p\right)=\frac{1}{Z(\sigma )}\exp\left(-\frac{d^{2}\left(\mu ,p\right)}{2\sigma^{2}}\right),$$

where the normalizing constant *Z* is independent from μ. This last fact is one of the motivations to use the above definition: the density computation does not require anything more than the geodesic distance evaluation. The basic facts about Gaussian-like distributions on symmetric spaces are given below.

**Definition** **1.** *A Riemannian symmetric space M is a complete Riemannian manifold on which geodesic symmetries exist everywhere and are isometric.*

The above geometric definition fits within a Lie theoretic construction. For the details on such objects, the reader can refer to [15] or for a more complete reference to [16].

**Definition** **2.** *A Riemannian symmetric space M is diffeomorphic to a quotient G/H where G is a Lie connected group and H is a compact Lie subgroup.*

The Lie group view enables the use of integral formulas [17] (pp. 203–209).

**Proposition** **1.** *Let M be a symmetric space of non-compact type, diffeomorphic to G/K and let g=h+a+n be the Iwasawa decomposition of the Lie algebra g. For any $f\in L^{1}\left(M\right)$:*

$$\int_{M}f\left(p\right)d\mathrm{vol}\left(p\right)=C\int_{H}\int_{a}f\left(\exp\left(\mathrm{Ad}(h)a\right)\cdot o\right)D\left(a\right)da\;dh,$$

*with da the Lebesgue measure on a, dh the normalized Haar measure on H and:*

$$D\left(a\right)=\prod_{\alpha \in \Sigma^{+}}\sinh^{m_{\alpha }}\left(|\alpha (a)|\right),$$

*where the product is taken over the set of positive roots $\Sigma^{+}$ and $m_{\alpha }$ is the dimension of the root space at α. o is a reference point.*

Applying the previous formula to the mapping:

$$\tilde{f}:p\mapsto \exp\left(-\frac{d^{2}\left(\mu ,p\right)}{2}\right)$$

with an origin at μ yields:

$$\int_{M}\tilde{f}\left(p\right)d\mathrm{vol}\left(p\right)=C\int_{H}\int_{a}\exp\left(-\frac{B(a,a)}{2\sigma^{2}}\right)D\left(a\right)da\;dh,$$

with *B* the Killing form. Since the Haar measure dh is normalized and the integrand does not depend on *h*, it comes:

$$\int_{M}\tilde{f}\left(p\right)d\mathrm{vol}\left(p\right)=Z\left(\sigma \right)=C\int_{a}\exp\left(-\frac{B(a,a)}{2\sigma^{2}}\right)D\left(a\right)da,$$

thus proving that the normalizing constant of the Gaussian-like distribution is independent from μ. Ref. [13] also proposes a maximum-likelihood estimator (MLE) suitable for the estimation of the parameters μ,σ.

**Proposition** **2.** *Let $X_{1},\ldots ,X_{n}$ be an iid sample drawn from the density $f_{\mu ,\sigma }$. The MLE estimator $\hat{\mu }$ (resp. $\hat{\eta }$) of μ (resp. $-1/2\sigma^{2}$) is the Riemannian barycentre of the sample (resp. ${\mathrm{argmax}}_{\eta }\eta \hat{\rho }-\log Z\left(\sigma \left(\eta \right)\right)$). In the expression of the $\hat{\eta }$ estimator, $\hat{\rho }$ is given by:*

$$\hat{\rho }=\frac{1}{N}\sum_{i=1}^{N}d^{(}\hat{\mu },X_{i}).$$

### 3.3. Wrapped Distributions

Among numerous properties, the Gaussian densities in $R^{d}$ are known to be solutions of the heat kernel. When the manifold of interest *M* is obtained as a quotient of a model space *H* by a discrete group, which is the case for example with Riemann surfaces, the heat kernel on *M* can be obtained by wrapping the one on *H* along the orbits of the group action.

The most basic distribution arising that way is the so-called wrapped Gaussian density on the unit circle in $R^{2}$. It is defined as:

(18) $$f_{wg}\left(\theta ;\sigma \right)=\frac{1}{\sqrt{2\pi }\sigma }\sum_{k\in Z}\exp\left(-\left(\theta +2k\pi \right)/\left(2\sigma^{2}\right)\right)$$

and clearly exhibits a period 2π. The parameter σ controls the concentration. It is worth noticing that $f_{wg}\left(\theta ;\sigma \right)$ is in fact the heat kernel on the circle. Evaluating the density involves finding the sum of a convergent series, which may be costly when the computation is done numerically. In the case of the wrapped Gaussian density, the very fast decay at infinity of the usual normal distribution limits the number of terms to be taken into account.

In [18], the heat kernel of simply connected Riemann surfaces is given by wrapping one of the only three possible model spaces: the Euclidean plane, the hyperbolic plane and the sphere. The respective heat kernels are:

(19) $$\begin{array}{c} K{\left(x,y,t\right)}_{R^{2}}=\frac{1}{4\pi t}\exp\left(-\frac{{∥x-y∥}^{2}}{4t}\right), \end{array}$$

(20) $$\begin{array}{c} K{\left(x,y,t\right)}_{H^{2}}=\frac{\sqrt{2}e^{-\frac{t}{4}}}{{\left(4\pi t\right)}^{3/2}}\int_{d(x,y)}^{\infty }\frac{se^{-\frac{s^{2}}{4t}}}{\sqrt{\cosh s-\cosh d(x,y)}}ds, \end{array}$$

(21) $$\begin{array}{c} K{\left(x,y,t\right)}_{S^{2}}=\frac{1}{4\pi }\sum_{n\in N}\left(2n+1\right)\exp\left(-n\left(n+1\right)t\right)P_{n}\left(\cos d_{S^{2}}\left(x,y\right)\right), \end{array}$$

where the expression for the hyperbolic plane comes from [19] (p. 360). The distances d(·,·) are the geodesic distances.

**Theorem** **1.** *Let M be a Riemann surface, U its universal cover and G its covering group. Let $K_{U}$ be the heat kernel on U. Then, the heat kernel on M is obtained by wrapping $K_{U}$ along the orbits of the covering group action:*

$$K_{M}\left(x,y,t\right)=\sum_{g\in G}K_{U}\left(\tilde{x},g\cdot \tilde{y},t\right),$$

*where $\tilde{x},\tilde{y}$ are fixed pre-images of respectively x,y for the covering map.*

The proof can be found in [18] (pp. 7–8). In principle, Theorem 1 yields a density similar to a directional one, but on a more general class of manifolds. Unfortunately, while a closed-form solution for the kernel $K_{U}$ is known, and is one of equations (19)–(21), the wrapped kernel is generally only computable numerically, after truncation to a finite number of terms in the sum.

In the case of surfaces with covering space $H^{2}$, it is possible to obtain a more convenient description. In this case, the genus g of the surface is strictly larger than 1, and the fundamental region in $H^{2}$ is a hyperbolic polygon with 4g sides. For any g∈G, its length is defined to be $l\left(g\right)=\inf_{x}d\left(x,gx\right)$, or using the conjugacy class of *g*: $l\left(g\right)=\inf_{x}d\left(x,kgk^{-1}\right)$ where *k* runs over *G*. Elements of *G* with non zero length are conjugate to hyperbolic elements in SL(2,R) and are thus conjugate to a scaling $x\mapsto \lambda^{2}x$. Furthermore, a conjugacy class represents a free homotopy class of closed curves, which contains a unique minimal geodesic whose length is l(g), where *g* is a representative element. For *p* a primitive element, let $G_{p}$ denote its centralizer in *G*. The conjugacy classes in *G* are all of the form $gp^{n}g^{-1},g\in G/G_{p}$ with *p* primitive and n∈Z. The wrapped kernel can then be rewritten as:

(22) $$K_{M}\left(x,y,t\right)=\sum_{p}\sum_{g\in G/G_{p}}\sum_{n\in Z}K_{H^{2}}\left(gx,p^{n}gy,t\right),$$

where *p* runs through the primitive elements of *G*. It indicates that the kernel $K_{M}$ can be understood as a sum of elementary wrapped kernels associated to primitive elements, namely those ${\tilde{k}}_{p}$ defined by:

(23) $${\tilde{k}}_{p}\left(x,y,t\right)=\sum_{n\in Z}K_{H^{2}}\left(x,p^{n}y,t\right),$$

with *p* primitive. Finally, *p* being hyperbolic, it is conjugate to a scaling, so it is enough to consider kernels of the form:

(24) $${\tilde{k}}_{p}\left(x,y,t\right)=\sum_{n\in Z}K_{H^{2}}\left(x,{\left(\lambda^{2}\right)}^{n}y,t\right),$$

with λ>1 a real number. To each primitive element *p*, a simple closed minimal geodesic loop is associated, which projects onto the axis of the hyperbolic transformation *p*. In the Poincaré half-plane model, such a loop unwraps onto the segment of the imaginary axis that lies between *i* and $i\lambda^{2}$. It is easily seen that the action of the elements $p^{n},n\in Z$ will give rise to a tiling of the positive imaginary axis with segments of the form $[\lambda^{2n},\lambda^{2(n+1)}[$. This representation allows a simple interpretation of the elementary wrapped kernels ${\tilde{k}}_{p}$, where the wrapping is understood as a winding.

Once again, the computational cost involved in the summation may be high. An approximation of the true wrapped kernel is given in [20]. It is similar to the vMF that approximates the wrapped Gaussian density in the circular case.

### 3.4. Exponential Families Arising from Group Actions

In many physical systems, some quantities must be invariant under the action of a group. This is a consequence of the celebrated Noether theorem [21]. Looking at little bit deeper at this theorem reveals the importance of a mapping, called the momentum map, that turns out to be constant under the evolution of the system. Turning back to densities but keeping the physical framework in mind, it seems natural to find families with a maximum entropy property, with constraints based on the momentum map. This approach and its relationship with information geometry has been thoroughly studied in [22] and will be presented later.

Another view at the same problem is to start from natural exponential families and try to impose group invariance [23]. Let *E* be a finite dimensional vector space and let $E^{*}$ be its dual.

**Definition** **3.** *The set E is the subset of positive Radon measures μ on E such that:*

- *μ is not concentrated on a proper affine subspace of E.*
- The set of the $\theta \in E^{*}$ such that:
  $$\int_{E}\exp\langle \theta ,x\rangle \mu \left(dx\right)<+\infty$$
  *has non empty interior, hereafter denoted Θ(μ).*

Any measure in E gives rise to a natural exponential family.

**Definition** **4.** *Let μ∈E. The natural exponential family with base measure μ is the parameterized family $P_{\theta },\theta \in \Theta \left(\mu \right)$ defined by:*

$$P_{\theta }\left(dx\right)=\exp\left(\langle \theta ,x\rangle -C_{\mu }\left(\theta \right)\right)\mu \left(dx\right).$$

Given a group *G* acting on *E*, a natural exponential family $P_{\theta },\theta \in \Theta \left(\mu \right)$ with base μ is said to be invariant if for any g∈G and any $p_{\theta }$, $g.p_{\theta }=p_{\theta^{\prime }}$ for a $\theta^{\prime }$ in Θ(μ). In the original work, groups of affinities were considered, namely: $g.x=A_{g}x+v_{g},x\in E$, where $A_{g}$ is the linear part of the affinity and $v_{g}$ the translation part. The main theorem characterizing the group action invariance for a given natural exponential family is [23]:

**Theorem** **2.** Let $P_{\theta },\theta \in \Theta \left(\mu \right)$ be a natural exponential family and G a group of affinities. The family $P_{\theta }$ is invariant under the action of G iff it exist an application $a:G\to E^{*}$ and an application b:G→R such that:

(25) $$\begin{array}{c} \forall \left(g,g^{\prime }\right)\in G\times G,\;a\left(gg^{\prime }\right)={}^{t}A_{g}^{-1}a\left(g^{\prime }\right)+a\left(g\right), \end{array}$$

(26) $$\begin{array}{c} \forall \left(g,g^{\prime }\right)\in G\times G,\;b\left(gg^{\prime }\right)=b\left(g\right)+b\left(g^{\prime }\right)-\langle a\left(g^{\prime }\right),A_{g}^{-1}v_{g}\rangle , \end{array}$$

(27) $$\begin{array}{c} \forall g\in G\;g\cdot \mu \left(dx\right)=\exp\left(\langle a(g),x\rangle +b(g)\right)\mu \left(dx\right). \end{array}$$

When *G* is a Lie group, a,b are differentiable mappings. With a group theoretic view, *a* is a cocycle for the action $g:x\in E^{*}\mapsto g\cdot x={}^{t}g^{-1}\cdot x$. Theorem 2 was given in [24] and improved in [23] (p. 4). As an example of use, natural exponential families on $R^{d}$ are characterized by the next theorem.

**Theorem** **3.** *A natural exponential family on $R^{d}$ is invariant under the action of SO(d) iff it admits as base measure $\mu =c\delta_{0}+\phi \left(\nu ⊗\sigma \right),$ where c≥0, $\delta_{0}$ is the delta measure at the origin, σ is the surface measure on $S^{d-1}$ and:*

- *ν is a measure on [0,+∞], with ν([0,1])<+∞ and it exists k>0 such that:*
  $$\int_{\left[1,+\infty \right]}x^{-(d-1)/2}\exp\left(kx\right)\nu \left(dx\right)<+\infty ,$$
- *$\phi :[0,+\infty ]\times S^{d-1}\to R^{d}-\left\{0\right\}$ is the polar coordinates mapping: (r,u)↦ru.*

Going back to the mechanical formalism and the momentum map, invariant exponential families can be put into a wider framework. The underlying object is a symplectic manifold (M,ω) where ω is a closed, non degenerate two-form on *M*. Let *G* be a connected Lie group acting on *M*.

**Definition** **5.** *The action of G on M is said to be symplectic if for all g∈G, g·ω=ω.*

The group action defines canonical vector fields on TM.

**Definition** **6.** *Let ξ∈g. The vector field $X_{\xi }$ is defined by:*

$$X_{\xi }:x\in M\mapsto {\left.\frac{d}{dt}\right|}_{t=0}\exp_{e}\left(t\xi \right)\cdot x,$$

*where e denotes the identity of G.*

The vector field $X_{\xi }$ can be interpreted as the infinitesimal action of *g* on the points of *M*.

**Definition** **7.** A mapping $U:M\to g^{*}$ is said to be a momentum map for the G-action if for all ξ∈g:

$$d\alpha_{\xi }=X_{\xi }┘\omega ,$$

*where $\alpha_{\xi }$ is the 0-form defined as:*

$$\forall x\in M,\;\alpha_{\xi }\left(x\right)=\langle U\left(x\right),\xi \rangle .$$

As noticed by Souriau [25], the momentum map allows a definition of exponential densities.

**Definition** **8.** *Let (M,ω) be a symplectic manifold of dimension 2d. The 2d-form $\omega^{n}/n!$ is a volume form on M, called the Liouville form.*

For a symplectic manifold, the Liouville form is the canonical one and will be denoted vol as in the Riemannian case. It is invariant by symplectomorphisms, which is a key ingredient in the definition of exponential families on *M*.

**Definition** **9.** *Let (M,ω) be a symplectic manifold. Let G be a connected Lie group acting on M with momentum map U. If the set of ξ∈g such that:*

$$\int_{M}\exp\left(-\langle U(x),\xi \rangle \right)d\mathrm{vol}\left(x\right)<+\infty$$

*has non empty interior, hereafter denoted by* Ξ*, the exponential family associated with the group action is defined to be:*

$$\forall \xi \in \Xi ,\;P_{\xi }\left(d\mathrm{vol}\left(x\right)\right)=\exp\left(-\langle U(x),\xi \rangle +C(\xi )\right)d\mathrm{vol}\left(x\right).$$

The momentum map may be used to define the analog of the usual moments and will in turn allow constraints definition in a maximum entropy approach.

**Definition** **10.** *With the hypothesis of Definition 9, the nth moment of a probability density f on M is defined as:*

$$E_{n}\left(f\right)=\int_{M}U{⊗}^{n}\left(x\right)f\left(x\right)d\mathrm{vol}\left(x\right).$$

Following [25], the exponential distributions are maximal entropy ones. Assuming that the first and second moments are defined for the exponential family, the next proposition holds.

**Proposition** **3.** Under the assumptions of Definition 9, the exponential distributions are the one with the largest entropy under the constraint $E_{n}=K$, with K a fixed vector in $g^{*}$.

The Souriau approach to invariant Gibbs measure has the obvious advantage of being intrinsic and adapted to a given group action. The parameters of the exponential families are elements of the Lie algebra and must be understood as a general way of fixing a generalized location (please note that it encompasses ’scale’ parameters also). It requires a symplectic base manifold, that is very natural in mechanics, but may be a little bit tricky to obtain in a more general setting.

## 4. Non-Parametric Density Estimation by Projection

### 4.1. The Euclidean Case

The intuitive idea behind the projection approach to density estimation on measure set (E,F,μ) is to use an orthonormal Hilbert basis ${\left(\phi_{n}\right)}_{n\in N}$ of the space $L^{2}\left(E,\mu \right)$ to construct an approximation of the unknown density $f\in L^{2}\left(E,\mu \right)$ from its projections. Namely,

(28) $$\alpha_{n}=E_{f}\left[\phi_{n}\left(X\right)\right]=\int_{E}\phi_{n}\left(x\right)f\left(x\right)d\mu \left(x\right),$$

where $E_{f}$ denotes expectation taken with respect to the density *f*. The reconstruction formula in $L^{2}\left(E,\mu \right)$ then reads as:

(29) $$f:x\mapsto \sum_{n\in N}\alpha_{n}\phi_{n}\left(x\right).$$

To turn the expansion into a density estimator, it is necessary to have an estimator of the coefficients $\alpha_{n}$. Furthermore, in applications, the series (29) has to be truncated to a finite number of terms. It is thus advisable to have a fast decay of the expansion coefficients when *n* goes to infinity.

For the first point, an empirical estimator of the expectation is generally used. Assuming an iid sample ${\left(X_{i}\right)}_{i=1\ldots N}$, the *n*-th projection is estimated as:

(30) $${\hat{\alpha }}_{n}^{N}=\frac{1}{N}\sum_{i=1}^{N}\phi_{n}\left(X_{i}\right),$$

and the density estimator is

(31) $${\hat{f}}^{N}\left(x\right)=\sum_{i=1}^{N}{\hat{\alpha }}_{n}^{N}\phi_{n}\left(x\right).$$

Taking the expectation shows that the estimator is unbiased. It is worth to notice that the projection method does not use more than the measure space structure and is thus easy to use on many spaces, including manifolds. It is also very fast to evaluate provided the expansion functions are known and can be computed easily. It nevertheless suffers from two flaws:

- The estimated density ${\hat{f}}^{N}$ is not necessarily non-negative as the expansion functions are generally not.
- Depending on the underlying measure space, a countable Hilbert basis may not exist and, even if this holds, the expansion functions may not be expressed in a closed form.

Most of the usual Hilbert spaces are countable. However, the Besocovitch space $B^{2}$ of almost periodic functions [26] is a classical example for which an uncountable Hilbert basis exists.

### 4.2. The Riemannian Case

When the underlying measure space is a compact Riemannian manifold (M,g) of dimension *d*, equipped with its volume form, the Laplace–Beltrami operator Δ naturally gives rise to a suitable Hilbert basis of $L^{2}\left(M,\mathrm{vol}\right)$, which we will denote by $L^{2}\left(M\right)$ for short. Indeed, there exists a sequence ${\left(\lambda_{n},\phi_{n}\right)}_{n\in N}$ such that for all n∈N,

$$\Delta \phi_{n}=\lambda_{n}\phi_{n},$$

and the $\phi_{n}$’s form a Hilbert basis of $L^{2}\left(M\right)$. The seminal work [27] details the theory of non-parametric projection based estimation in this case. Unfortunately, only in a few cases are the eigenfunctions of Δ known, limiting the applicability of the method. In the sequel, the estimator ${\hat{f}}^{N}$ with expansion truncated to the *Q* lowest terms will be denoted as ${\hat{f}}_{Q}^{N}$. Two main theorems describe the behavior of the estimator.

**Theorem** **4.** *Let f be a density of class $C^{s}\left(M\right)$, with derivatives belonging to $L^{2}\left(M\right)$. For any integer I>0, there exist constants $A_{f},B_{f}$ such that, for all Q≥I,*

(32) $$E_{f}\left[{∥f-{\hat{f}}_{Q}^{N}∥}_{L^{2}\left(M\right)}^{2}\right]\leq A_{f}\frac{Q^{d/2}}{N}+B_{f}Q^{-s}.$$

The second theorem gives a $L^{\infty }$ rate:

**Theorem** **5.** *Let f be a density of class $C^{s}\left(M\right)$, s>d/2, with derivatives belonging to $L^{2}\left(M\right)$. For any integer I>0, there exist constants $A_{M},B_{f}$ such that, for all Q≥I,*

(33) $$E_{f}{\left[{∥f-{\hat{f}}_{Q}^{N}∥}_{L^{\infty }\left(M\right)}^{2}\right]}^{1/2}\leq A_{f}\frac{Q^{d/2}}{N^{1/2}}+B_{f}Q^{(d/2-s)/2}.$$

The proofs are quite technical and the interested reader may refer to [27] for the details.

When the eigenfunctions of the Laplacian are known, the method is quite effective, since the evaluation of the estimated density at a point does not depend on the sample size. In most cases, however, a closed form for the eigenfunctions is not available, thus limiting the practical usability of this estimator.

A possible workaround is to estimate the true eigenfunctions and eigenvalues from an approximate discrete problem. In the approach of [28], a weighted graph is constructed from a net of points on the manifold *M*, with weights given by a function of the geodesic distance between vertices. For the graph, extracting the eigenfunctions and eigenvalues boils down to a standard linear algebra problem and can thus be solved efficiently. The result of the procedure is a finite set of eigenvectors, which represent discrete measures on the manifold. The projection estimator in such a case yields a quantization (i.e., a discrete approximation) of the estimated measure. Going back to a density can be done using a smoothing procedure, or simply by turning the discrete approximation to a piecewise constant one. In both cases, evaluation at point requires the computation of the geodesic distance to all the samples, thus making the overall procedure far less efficient.

Finally, it is worth mentioning that Laplacian eigenfunctions and representations are closely related when the underlying space is a Lie group. The reader may refer to [29] for the special case SO(d).

## 5. Non-Parametric Kernel Estimation

Non-parametric estimation of densities is performed using a sum of elementary bell-shaped functions known as kernels most of the time. It was introduced in the 1960s by Parzen in its seminal work [30] and Rosenblatt [31].

### 5.1. The Euclidean Case

Assuming that the unknown probability density *f* is univariate, defined on R, it can be estimated using an iid sample $X_{i},i=1\ldots N$ as:

(34) $$\hat{f}:x\in R\mapsto \frac{1}{Nh}\sum_{i=1}^{N}K\left(\frac{x-X_{i}}{h}\right),$$

where $K:R\to R^{+}$ is a symmetric kernel, i.e., a measurable mapping verifying K(x)=K(−x) and integrating to 1,

$$\int_{R}K\left(x\right)dx=1.$$

The parameter *h* is a strictly positive real number called the bandwidth of the estimator. It controls the degree of smoothing, and has to be tuned to get the best compromise between smoothness and accuracy. Kernel density estimators are biased. Their bias can be controlled in the following way.

**Proposition** **4.** (35) $$\left|f\left(x\right)-E[\hat{f}\left(x\right)]\right|\leq \int_{R}K\left(u\right)\left|f(x)-f(x-hu)\right|du.$$

**Proof.**  Let *X* be a random variable with law the common law of the sample. Taking the expectation of $\hat{f}$ gives:

$$E\left[\hat{f}\left(x\right)\right]=\sum_{i=1}\frac{1}{Nh}E\left[K\left(\frac{x-X_{i}}{h}\right)\right]=\frac{1}{h}E\left[K\left(\frac{x-X}{h}\right)\right]=\frac{1}{h}\int_{R}K\left(\frac{x-y}{h}\right)f\left(y\right)dy.$$

Letting u=(x−y)/h,

$$\frac{1}{h}\int_{R}K\left(\frac{x-y}{h}\right)f\left(y\right)dy=\int_{R}K\left(u\right)f\left(x-hu\right)du$$

comes. Then, since the kernel integrates to 1,

$$\left|f\left(x\right)-E[\hat{f}\left(x\right)]\right|\leq \int_{R}K\left(u\right)\left|f(x)-f(x-uh)\right|du,$$

hence proving the claim.  □

When the density is Lipschitz and the kernel satisfies

$$\int_{R}K\left(u\right)\left|u\right|du<+\infty ,$$

the bound can be improved to:

$$\left|f\left(x\right)-E[\hat{f}\left(x\right)]\right|\leq Ch\int_{R}K\left(u\right)\left|u\right|du,$$

where *C* is the Lispchitz constant. As expected, the bias vanishes as *h* goes to 0 but is non zero when h>0. The variance of the estimator can be computed pretty much the same way. Since the $X_{i}$’s are independent,

$$\mathrm{var}\;\left(\frac{1}{Nh}\sum_{i=1}^{N}K\left(\frac{x-X_{i}}{h}\right)\right)=\frac{1}{Nh^{2}}\mathrm{var}\;K\left(\frac{x-X}{h}\right)$$

comes. Using f(x−hu)=f(x−hu)−f(x)+f(x), we have:

$$\begin{array}{cc} \frac{1}{Nh^{2}}E\left[K{\left(\frac{x-X}{h}\right)}^{2}\right] & =\frac{1}{Nh}\int_{R}K^{2}\left(u\right)f\left(x-hu\right)du\\ & =\frac{1}{Nh}\int_{R}K^{2}\left(u\right)\left(f(x-hu)-f(x)\right)du+\frac{f(x)}{Nh}\int_{R}K^{2}\left(u\right)du. \end{array}$$

Thus, with the above Lipschitz condition,

$$\frac{1}{Nh^{2}}E\left[K{\left(\frac{x-X}{h}\right)}^{2}\right]\leq \frac{C}{N}\int_{R}K^{2}\left(u\right)\left|u\right|du+\frac{f(x)}{Nh}\int_{R}K^{2}\left(u\right)du.$$

It then appears that the upper bound of the variance of $\hat{f}$ goes to infinity as *h* goes to 0 due to the term

$$\frac{f(x)}{Nh}\int_{R}K^{2}\left(u\right)du.$$

This fact is an expression of the bias-variance dilemma.

When the density *f* is of class $C^{2}$, the bias can be expressed more conveniently as:

$$b\left(x\right)=f\left(x\right)-E\left[\hat{f}\left(x\right)\right]=-\frac{h^{2}}{2}f^{\left(2\right)}\left(x\right)\int_{R}K\left(u\right)u^{2}du,$$

provided

$$V_{K}=\int_{R}K\left(u\right)u^{2}du<+\infty .$$

Please note that $V_{K}$ is the variance of the kernel, considered as a probability distribution. It is further assumed in the sequel that the kernel is square summable. The following holds:

$$\mathrm{var}\;K\left(\frac{x-X}{h}\right)=h\int_{R}K^{2}\left(u\right)f\left(x-uh\right)du-h^{2}{\left(\int_{R}K\left(u\right)f\left(x-hu\right)du\right)}^{2},$$

so that the variance of the kernel estimator is

(36) $$\mathrm{var}\;\hat{f}\left(x\right)=\frac{1}{Nh}\int_{R}K^{2}\left(u\right)f\left(x-uh\right)du-\frac{1}{N}{\left(\int_{R}K\left(u\right)f\left(x-hu\right)du\right)}^{2}.$$

The mean square error (MSE) of the estimator $\hat{f}$ is the sum of the variance and the squared bias:

(37) $$E\left[{\left(\hat{f}\left(x\right)-f\left(x\right)\right)}^{2}\right]=\frac{1}{Nh}\int_{R}K^{2}\left(u\right)f\left(x-uh\right)du-\frac{1}{N}{\left(\int_{R}K\left(u\right)f\left(x-hu\right)du\right)}^{2}+\frac{h^{4}}{4}{\left[f^{\left(2\right)}\left(x\right)V_{K}\right]}^{2}.$$

The asymptotic MSE is thus:

(38) $$E\left[{\left(\hat{f}\left(x\right)-f\left(x\right)\right)}^{2}\right]\underset{N\to \infty ,\;h\to 0}{∼}\frac{f(x)}{Nh}\int_{R}K^{2}\left(u\right)du+\frac{h^{4}}{4}{\left[f^{\left(2\right)}\left(x\right)V_{K}\right]}^{2}.$$

There exists an optimal value of *h*, minimizing the previous expression,

$$h_{opt}={\left(\frac{{f\left(x\right)∥K∥}_{2}^{2}}{4NA}\right)}^{1/5},$$

where

$$A={\left[f^{\left(2\right)}\left(x\right)V_{K}\right]}^{2}.$$

This relation is very classical in density estimation and yields a pointwise convergence rate in $o(n^{-4/5})$, slower than the usual $o(n^{-1})$ in the parametric case.

In the multivariate case, two approaches are of common use. In the first one, the multivariate kernel in $R^{d}$ is just an *d*-fold tensor product of univariate kernels:

$$K\left(x_{1},\ldots ,x_{d}\right)=\prod_{i=1}^{d}K\left(x_{i}\right).$$

The tensor product kernel integrates to 1 by Fubini’s theorem; and the density estimator writes as:

(39) $$f\left(x\right)=\frac{1}{h^{d}}\sum_{i=1}^{N}K\left(\frac{x-X_{i}}{h}\right),$$

where $x\in R^{d}$. Apart from the slower convergence rates, things are similar to the univariate case.

Another option is to let the kernel depend on the norm of the difference between *x* and the $X_{i}$. In such a case, starting again with a univariate kernel *K*, the density estimator is:

(40) $$f\left(x\right)=\frac{C}{h^{d}}\sum_{i=1}^{N}K\left(\frac{∥x-X_{i}∥}{h}\right),$$

where *C* is a normalizing constant, such that:

$$C^{-1}=\int_{R^{d}}K\left(∥u∥\right)du.$$

In this framework, the multivariate kernel pertains to the class of radial basis functions, which has been thoroughly studied in approximation theory [32].

### 5.2. The Riemannian Case

Extending the previous derivations to manifolds is not direct, since several problems must be addressed. In the case of Riemannian manifolds, the geodesic distance can be used in place of the Euclidean norm, thus making the radial basis kernels more natural than the tensor product ones. A density estimator based on that is due to Pelletier [33]. His approach is briefly described in the sequel.

Let (M,g) be an orientable Riemannian manifold of dimension *d*, equipped with its volume form vol, and let $K:R_{+}\to R_{+}$ be a mapping such that:

(41a) $$\begin{array}{c} \int_{R_{+}^{d}}K\left(∥u∥\right)du=1, \end{array}$$

(41b) $$\begin{array}{c} \int_{R_{+}^{d}}u_{j}K\left(∥u∥\right)du=0,\;j=1\ldots d, \end{array}$$

(41c) $$\begin{array}{c} \int_{R_{+}^{d}}{∥u∥}^{2}K\left(∥u∥\right)du<+\infty , \end{array}$$

(41d) $$\begin{array}{c} t>1\Rightarrow K(t)=0, \end{array}$$

(41e) $$\begin{array}{c} \underset{t\in R^{+}}{\sup}K\left(t\right)=K\left(0\right). \end{array}$$

The main problem is to define what a radial basis function is in the context of manifolds. A similar question was addressed in [34] for the definition of the Laplacian in radial coordinates. The idea is to use the exponential mapping $\exp_{p}$ at a fixed point p∈M in a ball centered at the origin in $T_{p}M$ and of radius less than the injectivity radius r>0 at *p* and to compose *K* with the distance function to *p* to get an equivalent of a radial basis function in $R^{d}$. However, the volume form is not invariant under the action of the exponential map unless the manifold is flat. In order to keep the integral of the kernel equal to 1, a multiplicative corrective term has to be added.

**Definition** **11.** *Let p∈M. For any v in $T_{p}M$, let $\gamma_{v}:t\mapsto \exp\left(\frac{tv}{∥v∥}\right)$ and let $w_{i}$, i=1…d, be Jacobi fields along γ such that $w_{i}\left(0\right)=0$, for all i=1…d, $Dw_{1}/dt\left(0\right)=v/∥v∥$ and $Dw_{i}/dt\left(0\right)$, i=1…d, forms an orthonormal basis of $T_{p}M$. The volume density function $\theta_{p}:T_{p}M\to R$ is defined as:*

$$\theta_{p}\left(v\right)={∥v∥}^{d-1}\det\left(w_{1}\left(∥v∥\right),\ldots ,w_{d}\left(∥v∥\right)\right).$$

*By abuse of notations, we will also write $\theta_{p}\left(x\right)=\theta_{p}\left(\log_{p}x\right)$.*

The above definition with varying *p* extends readily to a mapping θ:TM→R. The exponential map $\exp_{p}:T_{p}M\to M$ induces by pullback a volume form $\exp_{p}^{*}\mathrm{vol}$ on $T_{p}M$, and it is worth noticing that $\theta_{p}$ is its density with respect to the Lebesgue measure of the Euclidean structure on $T_{p}M$. That is, in normal coordinates,

(42) $$d\exp_{p}^{*}\mathrm{vol}\left(x\right)=\theta_{p}\left(x\right)dx.$$

**Definition** **12.** *Let K be a kernel function in the sense of equation* (41) *and R>0 be the injectivity radius of M. The radial kernel at p∈M with bandwidth 0<r<R is defined as the mapping $K_{p,r}$:*

$$K_{p,r}:x\in M\mapsto \frac{1}{r^{d}}\frac{1}{\theta_{p}\left(x\right)}K\left(\frac{d(p,x)}{r}\right).$$

Since *K* has a support in [0,1] by assumption, the above expression will vanish for x∉B(p,r), where B(p,r) is the geodesic ball of center *p* and radius *r*. We will thus never have to bother about a possible vanishing of θ.

**Proposition** **5.** *The radial kernel satisfies:*

$$\int_{B(p,r)}K_{p,r}\left(x\right)d\mathrm{vol}=1.$$

**Proof.**  In normal coordinates at *p* and from (42), the following comes:

(43) $$\begin{array}{cc} \int_{B\left(p,r\right)=\exp_{p}B\left(0,r\right)}K_{p,r}\left(x\right)d\mathrm{vol} & =\int_{B(0,r)}K_{p,r}\left(\frac{∥x∥}{r}\right)\exp_{p}^{*}d\mathrm{vol}\\ & =\int_{B(0,r)}\frac{1}{r^{d}}K\left(\frac{∥x∥}{r}\right)dx_{1}\wedge \ldots \wedge dx_{d}=1. \end{array}$$

□

The next proposition in [33] shows that the kernel $K_{p,r}$ is centered at *p* in a probabilistic sense.

**Proposition** **6.** *Let p∈M and δ be the supremum of the sectional curvatures of M. Let μ be a probability measure, absolutely continuous with respect to the measure vol, and admitting $K_{p,r}$ as density. If $r<{\mathrm{inj}}_{p}\left(M\right)/2$ and, when δ>0, also $r<\frac{\pi }{4\sqrt{\delta }}$ then p is the unique minimizer of the function*

$$E:x\mapsto \int_{M}d^{2}\left(x,p\right)d\mu \left(p\right).$$

The proof can be found in [33] (Propostion 2.2, p. 5) and relies on a computation of the gradient of *E* and the convexity of a geodesic ball of radius *r* satisfying the above conditions. Based on the above results, it is natural to choose the following definition for the Riemannian kernel estimator.

**Definition** **13.** *Let $X_{i}$, i=1…N, be an iid sample on the manifold M with common density function f. Let 0<r<inj(M). The kernel density estimator on N points of f with kernel K and bandwidth r is:*

(44) $${\hat{f}}_{n,K,r}\left(x\right)=\frac{1}{N}\sum_{i=1}^{N}K_{x,r}\left(X_{i}\right).$$

Using Propostion 5, it is easy to see that ${\hat{f}}_{n,K,r}$ is a probability density on *M*. The estimator ${\hat{f}}_{n,K,r}$ is consistent and behaves roughly as in the Euclidean case.

**Theorem** **6.** *Let f be a $C^{2}$ probability density on M with bounded first and second derivative. If 0<r<inj(M), then there exists a constant $C_{f}$ such that:*

$$E_{f}\left[{\left|{\hat{f}}_{n,K,r}-f\right|}_{L^{2}\left(M\right)}^{2}\right]\leq C_{f}\left(\frac{1}{Nr^{d}}+r^{4}\right).$$

The proof that will be given here differs slightly from the one given in [33], and relies on the next lemma.

**Lemma** **1.** *Let γ:[0,1]→M be a smooth curve of M and let f:M→R be of class $C^{k+1}$. Letting $p=\gamma \left(0\right),u=\gamma^{\prime }\left(0\right)$, the Taylor expansion at order k of f along γ with integral remainder at p is given by:*

$$f\left(\gamma (t)\right)=f\left(p\right)+\nabla_{u}ft+\ldots \nabla_{u}^{k}f\frac{t^{k}}{k!}+\int_{0}^{t}\nabla_{\gamma^{\prime }\left(y\right)}^{k+1}f\frac{{\left(1-y\right)}^{k}}{k!}dy,$$

*where* ∇ *is an affine connection and $\nabla_{u}f=u\left(f\right)=df_{p}\cdot u$.*

**Proof.**  The mapping α=f∘γ is defined from [0,1] to R and is of class $C^{k+1}$. It thus admits a Taylor expansion with integral remainder

$$\alpha \left(t\right)=f\left(p\right)+\sum_{i=1}^{k}\frac{d^{i}\alpha \left(0\right)}{dt^{i}}\frac{t^{i}}{i!}+\int_{0}^{t}\frac{d^{k+1}\alpha \left(y\right)}{dt^{k+1}}\frac{{\left(1-y\right)}^{k}}{k!}dy.$$

For a smooth function ϕ:M→R, $\frac{d}{dt}\phi \circ \gamma \left(t\right)=\nabla_{\gamma^{\prime }\left(t\right)}\phi =d\phi \left(t\right)\cdot \gamma^{\prime }\left(t\right)$ and the result follows by induction.  □

**Lemma** **2.** *Let γ:[0,1]→M be a geodesic of M and f:M→R be of class $C^{k+1}$. Then, the remainder in Lemma 1 is upper bounded by*

$$C_{f}\ell {\left(\gamma \right)}^{k+1}\frac{1}{(k+1)!},$$

*where $C_{f}$ is a constant depending on f and ℓ(γ) is the length of γ.*

**Proof.**  Let $\omega \in T^{*}M$. Then, $\nabla_{\gamma^{\prime }}\omega \cdot \gamma^{\prime }=\left(\nabla_{\gamma^{\prime }}\omega \right)\cdot \gamma^{\prime }+\omega \cdot \nabla_{\gamma^{\prime }}\gamma^{\prime }=\left(\nabla_{\gamma^{\prime }}\omega \right)\cdot \gamma^{\prime }$. Proceeding by induction

$$\nabla_{\gamma^{\prime }}^{k+1}f=\left(\nabla_{\gamma^{\prime }\left(y\right)}^{k}df\cdot \gamma^{\prime }\right)=\left(\nabla_{\gamma^{\prime }\left(y\right)}^{k}df\right)\cdot \gamma^{\prime }.$$

The term $\nabla_{\gamma^{\prime }\left(y\right)}^{k}df$ is *k*-linear in $\gamma^{\prime }$ and involves derivatives of *f* up to order k+1 along γ. Since *f* is of class $C^{k+1}$ by assumption, it comes

$$\left|\nabla_{\gamma^{\prime }}^{k+1}f\right|\leq C_{f}{∥\gamma^{\prime }∥}^{k+1}.$$

Since γ is a geodesic, $∥\gamma^{\prime }∥=d$, where *d* is the length of γ. The claim follows by integration.  □

**Proof** **of** **Theorem** **6.** The proof is essentially an adaptation of the Euclidean case. The bias at x∈M is given by

$$b\left(x\right)=\int_{B(x,r)}K_{x,r}\left(y\right)f\left(y\right)d\mathrm{vol}\left(y\right)-f\left(x\right),$$

and since the kernel integrates to 1,

$$b\left(x\right)=\int_{B(x,r)}K_{x,r}\left(y\right)f\left(y\right)-f\left(x\right)d\mathrm{vol}\left(y\right).$$

Using normal coordinates at *x*, it comes

$$b\left(x\right)=\int_{B(0,r)}\frac{1}{r^{d}}K\left(\frac{∥u∥}{r}\right)\left(f(\exp_{x}\left(u\right))-f\left(x\right)\right)du.$$

Using lemma 1, $f\left(\exp_{x}\left(u\right)\right)-f\left(x\right)=\nabla_{u}f\left(0\right)+R_{f}\left(u\right)$. In coordinates, $u=u^{i}\partial_{i}$ and by linearity of the connexion ∇ and symmetry assumption on the kernel

$$\int_{B(0,r)}\frac{1}{r^{d}}K\left(\frac{∥u∥}{r}\right)\nabla_{u}f\left(0\right)du=\sum_{i=1}^{d}\nabla_{\partial_{i}}f\left(0\right)\int_{B(0,r)}\frac{1}{r^{d}}K\left(\frac{∥u∥}{r}\right)u_{i}du=0.$$

Since the first and second derivatives of *f* are assumed to be bounded on *M*, it exists, by Lemma 2, a constant $A_{f}$ such that $\left|R_{f}\left(u\right)\right|\leq A_{f}{∥u∥}^{2}$, yielding

$$\left|b\left(x\right)\right|\leq A_{f}\int_{B(0,r)}\frac{1}{r^{d}}K\left(\frac{∥u∥}{r}\right){∥u∥}^{2}=A_{f}r^{2}\int_{B(0,1)}K\left(∥u∥\right){∥u∥}^{2}du.$$

When the manifold is of finite volume, then the integral of the squared bias over *M* is bounded by:

$$A_{f}^{2}r^{2}{\left(\int_{B(0,1)}K\left(∥u∥\right){∥u∥}^{2}du\right)}^{2}\mathrm{vol}\left(M\right).$$

The case of unbounded volume is still tractable provided the first and second derivatives of *f* are square-integrable over *M*. The computation of the variance is very close to what has been done in the Euclidean case. Since the kernel has vanishing first moment, the only term remaining in the variance is the integral of the squared kernel, which equals

(45) $$\mathrm{var}\left(p\right)=\int_{B(p,r)}K_{p,r}^{2}\left(x\right)f\left(x\right)d\mathrm{vol}=\frac{1}{r^{2d}}\int_{B(p,r)}\frac{1}{\theta_{p}^{2}\left(x\right)}K^{2}\left(\frac{d(p,x)}{r}\right)f\left(x\right)d\mathrm{vol}.$$

In normal coordinates around *p*,

$$\int_{B(p,r)}K_{p,r}^{2}\left(x\right)f\left(x\right)d\mathrm{vol}=\frac{1}{r^{d}}\int_{B0,1}\frac{1}{\theta_{p}\left(\exp_{p}\left(ru\right)\right)}K^{2}\left({∥u∥}^{2}\right)f\left(\exp_{p}\left(ru\right)\right)du.$$

In contrast with the bias computation, the $\theta_{p}$ does not cancel. If $\left(p,x\right)\mapsto \theta_{p}\left(p,x\right)$ is bounded below by a constant L>0 and using the fact that *K* is bounded above by K(0), the variance is bounded above by

$$\frac{K^{2}\left(0\right)}{Lr^{d}}\int_{B(0,1)}f\left(\exp_{p}\left(ru\right)\right)du.$$

Integrating the variance over *M* yields

$$\int_{M}\mathrm{var}\left(p\right)dvol\leq \frac{K^{2}\left(0\right)}{Lr^{d}}\mathrm{vol}\left(B\left(0,1\right)\right).$$

Summing the variance and the squared bias completes the proof.  □

It is worth noticing that, while the final result is essentially the same as in the Euclidean case, assumptions are somewhat stronger: the kernel used must be of compact support and the θ function has to be bounded below. Furthermore, bandwidth has to be small enough to ensure that the exponential mapping is one to one.

### 5.3. Computing the Kernel in the Riemannian Case

A important feature of the non-parametric kernel estimation in the Euclidean case is the ease of computation. Estimating the density at a given point is done easily using inner products and function evaluations (polynomials in most cases). Furthermore, when the kernel is compactly supported, it is quite simple to avoid summing vanishing terms: a k-d tree [35] structure can be used to allow a quick distance evaluation, thus excluding points that will not contribute to the estimator.

In the Riemannian case, evaluation the kernel $K_{r,p}$ at a point *x* requires much more computation. First of all, the geodesic distance from *x* to *p* must be found, along with the $\theta_{p}$ function. If a shooting algorithm is used for approximating d(x,p), the derivative of the exponential mapping, needed for $\theta_{p}$, is generally also computed: the overall cost is thus not really different from the geodesic distance evaluation alone. Nevertheless, unless a closed form for the geodesics is known, the computational cost associated with the process is much higher than with Euclidean data.

In some special cases, however, the $\theta_{p}$ function can be obtained in a closed form. It is the case for symmetric spaces, when the roots system of the base Lie group is known: in such a case, the integral formulas in [17] yields directly $\theta_{p}$.

## 6. Discrete Density Estimation through Quantization

Arguably the simplest form of estimate, one can choose for an unknown probability measure μ is a discrete density $\hat{\mu }$ with finite support, i.e., a linear combination of Dirac distributions

$$\hat{\mu }\left(x\right)=\frac{1}{n}\sum_{i=1}^{n}w_{i}\delta_{a_{i}},$$

with weights $w_{i},i=1,\ldots ,n$ that sum up to 1. Finding such an approximation is the purpose of quantization. The theory was originally developed for signal compression purposes in the middle of the 20th century, in order to find appropriate ways to discretize a signal. Quantization was founded for probability distributions on Euclidean spaces, but its generalization to Riemannian manifolds presents no particular difficulty (with the necessary assumptions), and so we will present it directly in that more general setting. For further details, we refer the reader to [2] or the survey paper [36].

### 6.1. Optimal Quantization

Let μ be a probability measure with compact support on a Riemannian manifold (M,g). We assume that *M* is complete, i.e., that the exponential map at *x* is defined on the whole tangent space $T_{x}M$. Then, by the Hopf–Rinow theorem, *M* is also geodesically complete, that is, any two points x,y∈M can be joined by a geodesic of shortest length and the geodesic distance is everywhere well defined. Optimal quantization addresses the problem of approximating a random variable *X* with distribution μ by a *quantized* version q(X) where *q* has an image Γ of cardinal at most *n*. More precisely, defining

$$Q_{n}=\left\{q:M\to \Gamma \subset M\;\mathrm{measurable},\;|\Gamma |\leq n\right\},$$

the optimal quantization problem is to find q∈Q that minimizes the $L^{p}$ distance between *X* and q(X), with the following error

(46) $$q^{*}=\underset{q\in Q_{n}}{\mathrm{argmin}}\;E_{\mu }\left[d{\left(X,q\left(X\right)\right)}^{p}\right].$$

A solution to the above minimization problem is called an optimal *n*-quantizer, and the minimum error is denoted by

$$e_{n,p}\left(\mu \right)=\underset{q\in Q_{n}}{\inf}E_{\mu }\left[d{\left(X,q\left(X\right)\right)}^{p}\right].$$

**Proposition** **7.** *The search for optimal n-quantizers can be limited to nearest-neighbor projections, i.e.,*

$$e_{n,p}\left(\mu \right)=\underset{\Gamma ,|\Gamma |=n}{\inf}E_{\mu }\left[d{\left(X,q_{\Gamma }\left(X\right)\right)}^{p}\right],$$

*where $q_{\Gamma }:M\to \Gamma =\left\{a_{1},\ldots ,a_{n}\right\}$ is given by*

$$q_{\Gamma }\left(x\right)=\sum_{i=1}^{n}a_{i}1_{C_{i}\left(\Gamma \right)}\left(x\right),\;x\in M,$$

*and $C_{i}\left(\Gamma \right)$ denotes the $i^{th}$ Voronoi cell associated with* Γ,

$$C_{i}\left(\Gamma \right)=\left\{x\in M,d\left(x,a_{i}\right)\leq d\left(x,a_{j}\right)\;\forall j\neq i\right\}.$$

**Proof.**  Any *n*-quantizer *q* of image Γ⊂M verifies for all x∈M, $d\left(x,q\left(x\right)\right)\geq \inf_{a\in \Gamma }d\left(x,a\right)$, with equality if and only if $q\left(x\right)={\mathrm{argmin}}_{a\in \Gamma }d\left(x,a\right)$. Therefore, the optimal quantizer is the projection to the nearest neighbor of Γ. Moreover, if |Γ|<n and |suppμ|≥n, one easily checks that *q* can always be improved, in the sense of criteria (46), by adding an element to its image. This means that an optimal *n*-quantizer has an image of exactly |Γ|=n points, and is of the given form.  □

The optimal approximation of *X* is then given by the image $\hat{X}=q_{\Gamma }\left(X\right)$, while the optimal approximation of its distribution μ is given by the pushforward

(47) $$\hat{\mu }={\left(q_{\Gamma }\right)}_{*}\mu =\sum_{i=1}^{n}f\left[C_{i}\left(\Gamma \right)\right]\delta_{a_{i}},$$

where the atoms $a_{1},\ldots ,a_{n}$ are chosen to minimize the *distorsion function*, simply obtained by evaluating the cost function of (46) at $q=q_{\Gamma }$,

(48) $$F_{n,p}\left(a_{1},\ldots ,a_{n}\right)=E_{\mu }\left\{\min_{1\leq i\leq n}d{\left(X,a_{i}\right)}^{p}\right\}=\int_{M}\min_{1\leq i\leq n}d{\left(x,a_{i}\right)}^{p}d\mu \left(x\right).$$

Notice that if we seek to approximate μ by a single point a∈M (i.e., n=1) with respect to an $L^{2}$ criteria (p=2), we retrieve the definition of the Riemannian center of mass, also called the Fréchet mean [37].

(49) $$\bar{x}=E_{\mu }\left(X\right)={\mathrm{argmin}}_{a\in M}\int_{M}d{\left(x,a\right)}^{2}d\mu \left(x\right).$$

It is worth noting that the optimal quantization problem coincides with the optimal transport problem of approximating μ by the closest discrete measure with at most *n* supporting points, with respect to the $L^{p}$–Wasserstein distance.

**Proposition** **8.** *Let $P_{n}$ denote the set of all measures ν on M with |suppν|≤n. Then,*

$$e_{n,p}\left(\mu \right)=\underset{\nu \in P_{n}}{\inf}W_{p}{\left(\mu ,\nu \right)}^{p},$$

*where $W_{p}$ denotes the Wasserstein distance of order p, i.e.,*

$$W_{p}\left(\mu ,\nu \right)=\underset{P}{\inf}\int_{M\times M}d{\left(y,z\right)}^{p}dP\left(y,z\right).$$

*Here, the infimum is taken over all measures P on M×M with marginals μ and ν.*

The proof in the Euclidean case can be found in [2], and it applies verbatim to measures on manifolds. We restitute it here for the sake of completeness.

**Proof.**  Let q∈Q and f:M×M→M, f(x)=(x,q(x)) and $g:M\times M\to R_{+}$, $g\left(y,z\right)=d{\left(y,z\right)}^{p}$. Then, by definition of the image measure $f_{*}\mu$, we have

$$E_{\mu }\left[d{\left(X,q\left(X\right)\right)}^{p}\right]=E_{\mu }\left[g\circ f(X)\right]=E_{\left(f_{*}\mu \right)}\left[g(Y,Z)\right]=\int_{M}d{\left(y,z\right)}^{p}d\left(f_{*}\mu \right)\left(y,z\right).$$

Noticing that $\int_{y\in M}\left(f_{*}\mu \right)\left(dy,dz\right)=\mu \left(q^{-1}\left(dz\right)\right)$ and $\int_{z\in M}\left(f_{*}\mu \right)\left(dy,dz\right)=\mu \left(dy\right)$, i.e., that $\left(f_{*}\mu \right)$ has marginals *q* and $q_{*}\mu$, we get

$$e_{n,p}\left(\mu \right)\geq \underset{q\in Q_{n}}{\inf}W_{p}{\left(\mu ,q_{*}\mu \right)}^{p}\geq \underset{\nu \in P_{n}}{\inf}W_{p}{\left(\mu ,\nu \right)}^{p}.$$

On the other hand, if $\nu \in P_{n}$ has support $\Gamma =\{a_{1},\ldots ,a_{n}\}$, and *P* has marginals μ and ν, then

$$\begin{array}{cc} \int_{M\times M}d{\left(y,z\right)}^{p}P\left(dy,dz\right) & =\int_{M\times \Gamma }d{\left(y,z\right)}^{p}P\left(dy,dz\right)\\ & \geq \int_{M\times \Gamma }\min_{1\leq i\leq n}d{\left(y,a_{i}\right)}^{p}P\left(dy,dz\right)=\int_{M}\min_{1\leq i\leq n}d{\left(y,a_{i}\right)}^{p}\mu \left(dy\right), \end{array}$$

which gives $W_{p}{\left(\mu ,\nu \right)}^{p}\geq F_{n,p}\left(a_{1},\ldots ,a_{n}\right)$ and finally

$$\underset{\nu \in P_{n}}{\inf}W_{p}{\left(\mu ,\nu \right)}^{p}\geq \underset{\left(a_{1},\ldots ,a_{n}\right)}{\inf}F_{n,p}\left(a_{1},\ldots ,a_{n}\right)=e_{n,p}\left(\mu \right).$$

□

An important question that arises now is the existence of a minimizer of (48). The proof of the following claim can be found in [38].

**Proposition** **9.** *Let M be a complete Riemannian manifold and μ a probability distribution on M with density and a compact support. Then, the distorsion function $F_{n,p}$ is continuous and admits a minimizer.*

The minimizer $\alpha =(a_{1},\ldots ,a_{n})$, referred to as *optimal n-centers*, is in general not unique: any symmetry of μ, if it exists, will transform a minimizer into another minimizer of $F_{n,p}$. For example, any rotation of the optimal *n*-centers of the uniform distribution on the sphere conserves optimality.

The second question that comes naturally is: how does the error $e_{n,p}\left(\mu \right)$ one makes by approximating μ by (47) evolve when the number *n* of points grows? In the vector case, Zador’s theorem [2] (Theorem 6.2) tells us that it decreases to zero as $n^{-p/d}$, and that the limit of $n^{p/d}e_{n,p}\left(\mu \right)$ is proportional to the *$p^{th}$ quantization coefficient*, i.e., the limit (which is also an infimum) when μ is the uniform distribution on the unit square of $R^{d}$

$$C_{p}\left({\left[0,1\right]}^{d}\right)=\lim_{n\geq 1}n^{p/d}e_{n,p}\left\{U\left({\left[0,1\right]}^{d}\right)\right\}.$$

Moreover, when μ is absolutely continuous with density *h*, the asymptotic empirical distribution of the optimal *n*-centers is proportional to $h^{d/(d+p)}$.

In the case of a Riemannian manifold *M*, the moment condition of the flat case generalizes to a condition involving the curvature of *M*. The following term measures the maximal variation of the exponential map at x∈M when restricted to a (d−1)-dimensional sphere $S_{\rho }\subset T_{x}M$ of radius ρ

$$A_{x}\left(\rho \right)=\underset{v\in S_{\rho },w\in T_{v}S_{\rho },∥w∥=\rho }{\sup}∥d_{v}\exp_{x}\left(w\right)∥.$$

The following generalization of Zador’s theorem to Riemannian quantization was proposed by Iacobelli [39] (Theorem 1.4 and Corollary 1.5).

**Theorem** **7.** *Let M be a complete Riemannian manifold without boundary, and let $\mu =h\;d\mathrm{vol}+\mu_{s}$ be a probability measure on M, where dvol denotes the Riemannian volume form and $\mu_{s}$ the singular part of μ. Assume there exist $x_{0}\in M$ and δ>0 such that*

$$\int_{M}d{\left(x,x_{0}\right)}^{p+\delta }d\mu \left(x\right)+\int_{M}A_{x_{0}}\left\{d{\left(x,x_{0}\right)}^{p}\right\}d\mu \left(x\right)<\infty .$$

*Then,*

$$\lim_{n\to \infty }n^{p/d}e_{n,p}\left(\mu \right)=C_{p}\left({\left[0,1\right]}^{d}\right){∥h∥}_{d/(d+p)},$$

*where ${∥\cdot ∥}_{r}$ denotes the $L^{r}$-norm. In addition, if $\mu_{s}=0$ and $\left(a_{1},\ldots ,a_{n}\right)$ are optimal n-centers, then*

$$\frac{1}{n}\sum_{i=1}^{n}\delta_{a_{i}}\overset{D}{⟶}\lambda h^{d/(d+p)}dx\;\mathrm{as}\;n\to \infty ,$$

*where $\overset{D}{\to }$ denotes convergence in distribution and λ is the appropriate normalizing constant.*

### 6.2. A Numerical Scheme

In practice, to compute the optimal *n*-centers $\alpha =(a_{1},\ldots ,a_{n})$ from potentially large, manifold-valued datasets, one can search for the critical points of the distorsion function. Assume that the only knowledge that we have of the probability measure μ that we want to approximate is through an online sequence of i.i.d. observations $X_{1},X_{2},\ldots$ sampled from μ. A classical algorithm used for quadratic (p=2) vector quantization, and easily generalized to the Riemannian setting, is the *Competitive Learning Vector Quantization* algorithm, a stochastic gradient descent method based on the differentiability of the distorsion function $F_{n,2}$.

**Proposition** **10.** *Let $\alpha =\left(a_{1},\ldots ,a_{n}\right)\in M^{n}$ be an n-tuple of pairwise distinct components and p>1. Then, $F_{n,p}$ is differentiable and its gradient in α is*

$${\mathrm{grad}}_{\alpha }F_{n,p}={\left(-p\int_{\overset{˚}{C_{i}}\left(\alpha \right)}{∥\overset{⟶}{a_{i}x}∥}^{p-1}\frac{\overset{⟶}{a_{i}x}}{∥\overset{⟶}{a_{i}x}∥}\;\mu \left(dx\right)\right)}_{1\leq i\leq n}\in T_{\alpha }M^{n},$$

*where $\overset{˚}{C_{i}}\left(\alpha \right)$ is the interior of the $i^{th}$ Voronoi cell of α and $\overset{⟶}{xy}:=\exp_{x}^{-1}\left(y\right)$ denotes the vector that sends x on y through the exponential map. In particular, the gradient of the quadratic distorsion function is given by*

(50) $${\mathrm{grad}}_{\alpha }F_{n,2}={\left(-2\int_{\overset{˚}{C_{i}}}\overset{⟶}{a_{i}x}\;\mu \left(dx\right)\right)}_{1\leq i\leq n}=-2{\left(E_{\mu }1_{\left\{X\in \overset{˚}{C_{i}}\right\}}\overset{⟶}{a_{i}X}\right)}_{1\leq i\leq n}.$$

The proof can be found in [38].

Notice that optimal *n*-centers are Riemannian centers of mass of their Voronoi cells, in the sense of (49). More generally, for any value of *p*, each $a_{i}$, i=1,…,n, is the *p*-mean of its Voronoi cell, i.e., the minimizer of

$$a\mapsto \int_{\overset{˚}{C_{i}}\left(\alpha \right)}d{\left(x,a\right)}^{p}\mu \left(dx\right).$$

Therefore, the optimal *n*-centers are always contained in the compact support of μ. Notice also that the opposite direction of the gradient is, on average, given by the vectors inside the expectation. Competitive learning quantization consists in following this direction at each step *k*, that is, updating only the center $a_{i}$ corresponding to the Voronoi cell of the new observation $X_{k}$, in the direction of that new observation. In the Riemannian setting, instead of moving along straight lines, we simply follow geodesics using the exponential map. This gives a convergent algorithm, as shown in [38], which is particularly adapted to large datasets as it is online, i.e., it processes one data point at a time. Moreover, unlike kernel based methods, it requires few distance computations: of order n×N instead of $N^{2}$ if *N* is the size of the dataset and n≪N the size of the summary.

Finding the right number of centers may be a difficult question when there is no a priori knowledge on the distribution to be approximated. In practice, it is mainly a trial and error procedure, unless the problem itself allows an initial guess of the value. An alternative approach is to use the fact that any quantization defines a clustering by Voronoï cells: the quality of the later can be used to assess the performance of the former. Quite a lot of standard indicators exist for that purpose [40]. Just to mention a simple one, the Silhouette [41] is easy to compute and does not require the knowledge of the membership labels.

## 7. Some Open Problems

Density estimation on manifolds is still an open area of research and as such has several questions that are not yet been answered. Some of them are given below, which may serve as a starting basis for further research.

### 7.1. Parametric Estimation and Symplectic Structure

A non-trivial aspect of the parametric estimation on manifold is that the parameter space is generally different from the base manifold. In fact, it is already the case for location-scale models in $R^{d}$, since the scale parameter is an element of $R^{+}$, but the underlying abelian group structure makes the location part an element of $R^{d}$, which is also the base space. If one tries to extend the concept of location parameters to manifolds, two approaches can be used:

- Use local coordinates as parameters, and mimic the vector case as in [9].
- Replace the abelian group underlying $R^{d}$ by a Lie group acting on the base manifold.

In view of the general results mentioned in Section 3.4 for exponential families defined on symplectic manifolds, the second setting is the most natural one, but is not as general as the first one. However, given a Riemannian manifold *M*, its cotangent bundle $TM^{*}$ has a canonical symplectic structure, obtained from the so-called tautological one form [42] (pp. 9–14).

**Definition** **14.** Let $\pi :TM^{*}\to M$ be the canonical projection and $d\pi :TTM^{*}\to TM$ its derivative. The tautological one-form $\alpha :TTM^{*}\to R$ is defined pointwise as:

$$\alpha_{\omega }\left(v\right)=\omega \left(d\pi v\right),$$

where $v\in TTM^{*}$ and $\tilde{\pi }v=\omega$, with $\tilde{\pi }:TTM^{*}\to TM^{*}$ the canonical projection.

The tautological one-form gives rise to a symplectic form:

**Proposition** **11.** The two-form ω=−dα provides $TM^{*}$ with a symplectic struture. In local coordinates $(x_{,}\ldots x_{d},\xi_{1},\ldots ,\xi_{d}),$ it reads as:

$$\omega =\sum_{i=1}^{d}dx_{i}\wedge d\xi_{i}.$$

Given the symplectic structure on $TM^{*}$, one can derive exponential families provided a group action by symplectomorphisms exists. This construction may be a starting point for a quite general definition of parameterized families on manifolds, by moving from the base manifold to its cotangent bundle. It is worth noticing that location models may also be constructed using generating functions [42].

### 7.2. Manifolds with Boundaries

All the density estimators presented before where defined on manifolds without boundaries. However, in several settings, boundaries arise naturally as limiting cases: as an example, the space of symmetric positive semi-definite matrices of dimension d×d has stratified boundaries, namely the manifolds of matrices of rank 0≤p<d. Densities localized on the boundaries are degenerate versions of the one defined in the interior, but must be taken into account as they carry a non-vanishing mass. Apart from the projection estimator that fits in the manifolds with boundaries framework, all the other methods must be adapted. In particular, distributions defined on matrix spaces must be parameterized in such a way that rank deficiency is allowed in the parameter space. For manifolds with corners [43], this is easily obtained from the particular structure of the local charts that map open subsets of the manifold to open subsets of $[0,+\infty [\times R^{d-k},0\leq k\leq d$ and it turns out that all examples of matrix manifolds fall within this frame.

Extending parameterized density estimators or kernel estimators to manifolds with corners will have many practical applications, especially when dealing with matrix statistics. The same applies to optimal quantization, where dirac densities located on the boundaries must be added to the initial model.

### 7.3. Constrained Quantization

In the Riemannian quantization problem, an approximate distribution in the form $\sum_{i=1}^{n}\mu_{i}\delta_{a_{i}}$, with $a_{1},\ldots a_{n}$ the optimal centers located on the base manifold *M* and $\mu_{1},\ldots ,\mu_{n}$ be positive real numbers summing to 1. While such a representation is very natural both from a optimal approximation and clustering point of view, some specific applications require putting additional constraints on the weights $\mu_{i},i=1\ldots n$ [44]. A common one is to impose that they are all equal, which in a clustering application means that all classes will have an equal expected number of members. The stochastic gradient algorithms presented above is no longer valid for dealing with this problem and has to be adapted. It is still an open question to find a suitable procedure.

## 8. Conclusions

Probability densities approximation and estimation on Riemannian manifolds are topics receiving an increasing interest in the statistics community. In many applications, data are living on non-Euclidean spaces and adapted procedures must be designed.

In Euclidean spaces, parametric and non-parametric estimation procedures have been intensively studied and are well understood. In the Riemannian manifold setting, several non-equivalent extensions are possible, making the task to find the right one quite tricky. The most obvious way of dealing with manifold valued data is to use a local linearization, like the use of normal coordinates. When applied to densities, it may be used to derive maximum entropy distributions with fixed moments, but some care must be taken with the cut locus, which may be charged by the defined distribution. Other approaches rely on a direct use of the geodesic distance, both in the parametric and non-parametric estimation framework. The computational cost associated with this operation may be high, as a differential problem with boundary conditions has to be solved. When dealing with kernel estimation, Jacobi fields must also be evaluated. While it involves only a classical Ordinary differential equation (ODE). integration, it is an increase in the overall complexity of the procedure.

Parametric estimation using exponential families based on group action invariance are theoretically appealing and makes use of the underlying information on the data. This a especially important when data is highly structured. A closed form momentum map is nevertheless a prerequisite to derive an efficient implementation.

Finally, projection based estimation is in principle very efficient, provided one can obtain the eigenfunctions of the Laplace–Beltrami operator in a closed form, or at least efficiently approximate them. In low dimension, numerical schemes may be used for that purpose, but do not scale well.

Classical directional densities and their generalizations are numerically appealing and offer a sound framework for some manifolds. They are designed on an ad hoc basis, and may not be adapted to all cases. Most of them are maximal entropy based and often exhibit a group invariance. An interesting question is whether approximate directional densities can be found for a wrapped distribution arising from a model heat kernel.

As a general conclusion, extending the usual distributions to general manifolds is by no way an elementary procedure. Furthermore, where in the Euclidean case some distributions satisfy many equivalent defining properties, it will not be the case for manifolds. Maximum entropy is generally a good criterion, provided the fixed moments are defined in a simple and natural way. Finally, an often overlooked issue with densities defined on Riemannian manifolds is the associated computational cost when closed forms are unknown. Since all distance computations require solving a boundary value problem, the complexity of the manifold algorithms may be some order of magnitudes higher than their Euclidean counterparts. This also limits the practical dimension of the problems.
